# Supplementary material for: Exploring the topology and dynamic growth properties of co-invention networks and technology fields
Source: PLoS One. 2021 Sep 2;16(9):e0256956. doi: 10.1371/journal.pone.0256956 (PMC8412278; doi:10.1371/journal.pone.0256956)
Supplement: Attachment — Submitted filename: Response to Reviewers_PLOS ONE_R.docx [file pone.0256956.s003.docx]

**Response to Referees Comments**

**PONE-D-20-41087**

**Exploring the topology and dynamic growth properties of co-invention networks and technology fields**

**PLOS ONE**

The authors would like to thank the Editor-in-Chief and the Referees for their time and effort in providing valuable comments and insights. We also appreciate the opportunity to improve our research and results. We agree with all the comments and we have revised our manuscript accordingly. We hope that modifications done allowed for significant improvement of the text clarity.

- ***Response to comments from Anonymous Reviewer #2***

*Reviewer #2: The authors made a great effort to answer the comments of the four reviewers and managed to improve their manuscript. Although I do not agree with some of the statements, I like this work and want it to be published. However, the Figures are unacceptable. First of all, the drawings are unintelligible and shall be redrawn, the font size shall be increased. Fig. 4 misses vertical axis, Fig. 6 misses plot legend, the plot legend of the Fig. 4 is outside the figure area. The most important problem is with the Figure captions. They are simply absent. This manuscript mixes Figure captions and Figure description/Discussion together. This is not good. The Figure caption shall be separate and tell to the readers what we see in the Figure, what is what. The reader shall understand the paper in grosso modo through looking at the figures and reading figure captions.*

Thanks for the comments. We agree that poor-quality images are unacceptable. In order to create high-quality images,

in this revised version of the paper, Figures are included separately as EDS image files as established in the figure preparation guidelines. We also ensure that all the images have a resolution of at least 300 pixels per inch and appear sharp, not pixelated.

*Fig. 1 - I do not understand anything here. There are no axes, no titles, no legend.*

A: We agree with the reviewer that we need to clarify Fig. 1. The paragraph has been revised as follows (pp. 17-18):

“Fig 1 visualizes the layout for the total and IPC technology networks using Cytoscape's algorithm [1]. The Cytoscape software is designed to visualize very large networks [2]. The central organizing principle of Cytoscape is a network graph, with inventors represented as nodes and interactions represented as links or edges between nodes. The network core refers to a central and densely connected set of network nodes (the largest connected component of the network), while the periphery of the network denotes the sparsely connected set of nodes, which are linked to the core. The non-connected or isolated nodes from the bulk of the network are shown to the side and bottom of the central figure”

For representation, network graphs are presented without axes. However, to facilitate the interpretation we included a brief text pointing to the core, periphery, and the non-connected set of nodes. We also improve the quality and color of the image submission, and include a new title: Visualization of the technology co-inventor networks using Cytoscape, 1999–2019.

*Fig.2 shows a fat-tail distribution of the number of the authors of the paper. I do not understand the importance of this indicator for the invention process. Do you mean that the large number of collaborators submitting a patent indicates a collective nature of the invention process?*

A: Fig. 2 presents a histogram of the distribution of inventors according to the number of connections that they have. Data for 1999, 2000 and 2019 are plotted to investigate the inter-temporal stability of this network [3]. In particular, Fig. 2 presents for 2019 the distribution of the 1.8 million vertices (inventors) according to the number of connections (degree) that they have presented (6.7 million edges or links in total). For the total network in 2019, there are 267.626 inventors with one link, and one inventor with 1.346 links. The power law (the straight line in a log x log scale) behavior of the distribution of connections (links) indicates that the co-inventorship collaboration network is a scale-free one. Power-law-like distributions for degrees state that there exist many low degree nodes (a large number of nodes with not so many connections), whereas only a few high degree nodes (a small number of nodes with a very high connection) in real graphs. [4]. However, as discussed in the paper (pp.19-20):

“An interesting fact is that some of the graphs will exhibit power-law properties over the years, whereas others will not. For instance, the total network does not exhibit a scale-free range in the degree distribution in 1999. This can be explained because our empirical data contain only those nodes and links that have been created that year. By 2009 (as displayed in Fig 2), the distribution is already power-law ($\gamma=4.37)$ and will stay the same in subsequent years. Section C is the only network that exhibits power-law distributions in both 2009 and 2019. Section A shows power-law behavior in 2009 but not in 1999 or 2019. Compared to the other distributions (and with an average confidence level of 99%), sectors A y H behave like an exponential, sectors D and E behave like a Poisson, and sector G behaves like a lognormal model. Therefore, and based on our data, we found indications of scale-free networks, but also significant deviations from an ideal power-law in some of these networks over time. Thus, the hypothesis H2, which states that the co-inventor collaboration network will follow a power-law regardless of the technological domain, is not supported by our analysis.”

*Fig. 3- With respect to the preferential attachment - I do not know what are the implications of the validity of this hypothesis with respect to the assessment of the invention process, but Fig. 3 clearly disqualifies this hypothesis.*

A: Fig 3 shows the vertex degree *k* value in the horizontal axis, and the attachment function estimate (α) in the vertical axis. The reviewer is correct: only the case of α = 1 gives rise to a perfect PA function. For 0 $<\alpha<1$ the resulting degree distribution exhibits sublinear PA, whereas for α > 1 superlinear attachment [5]. The paragraph has been revised as follows (pp. 20-21):

“Our measurements using aggregated data from the category field networks show that the growth of co-inventorships can be explained based on the organizing principle of PA, although the attachment mechanism deviates from an ideal power-law. Applying SNA tools, our data reveals a sublinear preferential attachment model with exponent $\alpha=0.67-0.72$. In the sublinear PA regime, new nodes (i.e., inventors) are connected to old ones with a probability proportional to a fractional power of their degree. In this case, the network becomes a gel-like where every node is connected to all other nodes and degree distribution is the stretched exponential rather than power-law [6]. This asymptotic degree distribution for sublinearly growing networks is characterized by an exponent smaller than one and a maximum degree that scales as a power of the logarithm of the number of nodes [7]. Therefore, our results from using large datasets support Hypothesis H3, that is, the sublinear regime, and this is consistent with previous measurements on scientific collaboration [6, 8]. This also indicates that a perfect PA function (that is, the chance that an inventor gets a new collaborator is proportional to their current number of collaborators) is not supported in our study.”

We also introduced the following text in the manuscript to briefly comment on this issue (pp. 26-27):

“We can infer that the observed distributions fit in a growth model in which the source of added edges are chosen according to a sublinear PA, but the destination is selected at random [9]. This is indicative of an inverse Matthew effect or a cumulative disadvantage and implies that past patent activity is not necessarily correlated with whatever growth mechanism is actually at play [10]. Therefore, we cannot assume that there is a clear growth mechanism for making new connections for inventors with a high degree [9].”

***Response to comments from Anonymous Reviewer #4***

*Reviewer #4: Thank you for your efforts in revision. The third and fourth points in the conclusion section were very meaningful. I think the value of the paper would be further enhanced if these (third and fourth points) were emphasized and stated. The first and second findings are relatively common, so I honestly don't think they are very important.*

A: Thanks, revised. We agree with the reviewer that strengthen our findings further. Following the reviewer’s suggestions, the following paragraph has been revised at the Results section as follows (pp. 20-21):

“In the sublinear PA regime, new nodes (i.e., inventors) are connected to old ones with a probability proportional to a fractional power of their degree. In this case, the network becomes a gel-like where every node is connected to all other nodes and degree distribution is the stretched exponential rather than power-law [6]. This asymptotic degree distribution for sublinearly growing networks is characterized by an exponent smaller than one and a maximum degree that scales as a power of the logarithm of the number of nodes [7]. Therefore, our results from using large datasets support Hypothesis H3, that is, the sublinear regime, and this is consistent with previous measurements on scientific collaboration [6, 8]. This also indicates that a perfect PA function (that is, the chance that an inventor gets a new collaborator is proportional to their current number of collaborators) is not supported in our study.”

The following paragraph has been changed at the Conclusion and Discussion section (pp. 26-27):

“Third, all the inventor networks considered here grew at a lower PA rate than in other collaboration networks such as scientific papers and patent citations [6]. We can infer that the observed distributions fit in a growth model in which the source of added edges are chosen according to a sublinear PA, but the destination is selected at random [9]. This is indicative of an inverse Matthew effect or a cumulative disadvantage and implies that past patent activity is not necessarily correlated with whatever growth mechanism is actually at play [10]. Therefore, we cannot assume that there is a clear growth mechanism for making new connections for inventors with a high degree [9]. It is also possible that the innovation system is not yet at full capacity in terms of the number of players necessary to make it sustainable or self-organizing [11]. Self-organization may be among the most important mechanisms leading to scale invariance and decreased system entropy, which are the foundations of evolution in complex systems [10]. This sublinear PA regime seems to resonate deeply with the expected restrictions on the behavior of inventors in a business setting, in which inventors are required to first seek the collaboration of (or are coerced to collaborate with) others within their firms and corporate alliances before accessing new knowledge outside their organizational boundaries [12]. Patent collaboration is a complex task and company policies seem to play a decisive role, especially in shaping the partner selection of inventor teams and therefore collaboration networks. This is increasingly the case as the increasing regulatory patent protection system tends to restrict rather than advance knowledge spillover and sharing [13].”

And also (pp. 28-29):

“The smallest technology groups, namely invention technologies in the fields of D. Textile and paper and E. Construction do not fit the gel point rules. Although there were indications of an early phase aggregation process, these inventor networks were still highly fragmented and much smaller in size than any of the other technology networks studied here, and this had important implications for their network topology. The creation of a complex structure as a result of the dynamics of the system is still pending in these sectors, as it is evident that the organization is still notably relegated in comparison with that of the other’s sectors.”

We hope that with the aforementioned changes we have addressed all issues mentioned in the reviewers’ comments.

Sincerely,

The authors.

**References**

1.	Shannon P, Markiel A, Ozier O, Baliga NS, Wang JT, Ramage D, et al. Cytoscape: a software environment for integrated models of biomolecular interaction networks. Genome Res. 2003;13(11):2498-504. doi: doi: 10.1101/gr.1239303.

2.	Kohl M, Wiese S, Warscheid B. Cytoscape: Software for Visualization and Analysis of Biological Networks. In: Hamacher M, Eisenacher M, Stephan C, editors. Data Mining in Proteomics: From Standards to Applications. Totowa, NJ: Humana Press; 2011. p. 291-303.

3.	Ribeiro LC, Rapini MS, Silva LA, Albuquerque EM. Growth patterns of the network of international collaboration in science. Scientometrics. 2018;114(1):159-79. doi: 10.1007/s11192-017-2573-x.

4.	McGlohon M, Akoglu L, Faloutsos C. Statistical Properties of Social Networks. In: Aggarwal CC, editor. Social network data analytics: Springer; 2011. p. 17-42.

5.	Newman ME. The Structure and Function of Complex Networks. SIAM Review. 2003;45(2):167 - 256. PubMed PMID: doi:10.1137/S003614450342480.

6.	Golosovsky M. Mechanisms of Complex Network Growth: Synthesis of the Preferential Attachment and Fitness Models. Physical Review E. 2018;97(6):062310. doi: 10.1103/physreve.97.062310.

7.	Jog V, Loh P. Analysis of Centrality in Sublinear Preferential Attachment Trees via the Crump-Mode-Jagers Branching Process. IEEE Transactions on Network Science and Engineering. 2017;4(1):1-12. doi: 10.1109/TNSE.2016.2622923.

8.	Wagner CS, Leydesdorff L. Network structure, self-organization, and the growth of international collaboration in science. Research Policy. 2005;34(10):1608-18.

9.	Newman ME, Forrest S, Balthrop J. Email networks and the spread of computer viruses. Physical Review E. 2002;66(3):035101.

10.	Katz JS. What Is a Complex Innovation System? PLOS ONE. 2016;11(6):e0156150. doi: 10.1371/journal.pone.0156150.

11.	Perc M. Growth and structure of Slovenia’s scientific collaboration network. Journal of Informetrics. 2010;4(4):475-82. doi: <https://doi.org/10.1016/j.joi.2010.04.003>.

12.	Crescenzi R, Nathan M, Rodríguez-Pose A. Do inventors talk to strangers? On proximity and collaborative knowledge creation. Research Policy. 2016;45(1):177-94.

13.	Ma Y, Chi Q, Song L. Revealing structural patterns of patent citation by a two-boundary network model based on USPTO data. IEEE Access. 2020;8:23324-35. doi: 10.1109/ACCESS.2020.2969654.
